# Roles for ADAM17 in TNF-R1 Mediated Cell Death and Survival in Human U937 and Jurkat Cells

**DOI:** 10.3390/cells10113100

**Published:** 2021-11-10

**Authors:** Jürgen Fritsch, Julia Frankenheim, Lothar Marischen, Timea Vadasz, Anja Troeger, Stefan Rose-John, Dirk Schmidt-Arras, Wulf Schneider-Brachert

**Affiliations:** 1 Department of Infection Prevention and Infectious Diseases, University Hospital of Regensburg, 93053 Regensburg, Germany; Julia.Frankenheim@stud.uni-regensburg.de (J.F.); Selina-Timea.Vadasz@stud.uni-regensburg.de (T.V.); Wulf.Schneider@klinik.uni-regensburg.de (W.S.-B.); 2 Department of Pediatric Hematology, Oncology and Stem Cell Transplantation, University Hospital of Regensburg, 93053 Regensburg, Germany; Lothar.Marischen@klinik.uni-regensburg.de (L.M.); Anja.Troeger@klinik.uni-regensburg.de (A.T.); 3 Institute of Biochemistry, Christian-Albrechts-Universität zu Kiel, 24105 Kiel, Germany; rosejohn@biochem.uni-kiel.de; 4 Department of Biosciences, Paris-Lodron-University Salzburg, 5020 Salzburg, Austria; dirk.schmidt-arras@plus.ac.at

**Keywords:** TNF-R1, ADAM17, cell death

## Abstract

Signaling via death receptor family members such as TNF-R1 mediates pleiotropic biological outcomes ranging from inflammation and proliferation to cell death. Pro-survival signaling is mediated via TNF-R1 complex I at the cellular plasma membrane. Cell death induction requires complex IIa/b or necrosome formation, which occurs in the cytoplasm. In many cell types, full apoptotic or necroptotic cell death induction requires the internalization of TNF-R1 and receptosome formation to properly relay the signal inside the cell. We interrogated the role of the enzyme A disintegrin and metalloprotease 17 (ADAM17)/TACE (TNF-α converting enzyme) in death receptor signaling in human hematopoietic cells, using pharmacological inhibition and genetic ablation. We show that in U937 and Jurkat cells the absence of ADAM17 does not abrogate, but rather increases TNF mediated cell death. Likewise, cell death triggered via DR3 is enhanced in U937 cells lacking ADAM17. We identified ADAM17 as the key molecule that fine-tunes death receptor signaling. A better understanding of cell fate decisions made via the receptors of the TNF-R1 superfamily may enable us, in the future, to more efficiently treat infectious and inflammatory diseases or cancer.

## 1. Introduction

Signaling via TNF-R1 evokes various biological outcomes, ranging from the induction of inflammation and gene transcription to the activation of different modes of cell death. Cell survival signaling via NFκB emanates from cell surface resident receptors, recruiting so-called TNF-R1 *complex I*. Cell death signaling requires *complex II* formation. Under physiological conditions, the activation of TNF-R1 favors *complex I* formation, instead of cell death induction, in most cell types. In some cell types, the activation of apoptosis requires receptor internalization and *complex II* recruitment in the death domain of TNF receptors. However, *complex II* can dissociate from the receptor and occurs as a soluble cytosolic complex. The mechanisms beyond this point are not fully understood. In contrast to apoptosis induction via *complex II* formation, necroptosis is a caspase-8 independent form of cell death. In cells in which caspase-8 is compromised by pharmacological inhibition or endogenous caspase-8 inhibitors such as c-FLIP isoforms, *complex II* is remodeled to promote necrosome formation, comprising RIPK1, RIPK3 and MLKL. RIPK3 phosphorylates MLKL, which triggers its oligomerization and pore formation in the plasma membrane, finally resulting in cell swelling and rupture [1,2].

Aberrant signaling via TNF-R1 results in excess inflammation, cancer formation and reduced infection defense. Despite years of intense research it is not fully understood how the diversification of signaling outcomes is regulated or what the molecular checkpoints are [1].

Several studies have linked receptor internalization to TNF induced apoptosis and demonstrated that the inhibition of TNF-R1 internalization impairs *complex II* formation and, finally, apoptosis induction in various cell lines, as well as in a mouse model [3,4,5,6]. One report showed that regulated intramembrane proteolysis (RIP) by γ-secretase is required for receptor internalization and apoptosis induction in MCF-7 cells [7]. In general, it is assumed that extracellular cleavage by proteases of the A disintegrin and metalloproteinase enzyme family (i.e., ADAM17 or ADAM10) precedes RIP. While ADAM10 activity appears to be constitutive, ADAM17 can be activated (i.e., by phorbol myristate acetate, PMA) [8]. In human umbilical vein endothelial cells (HUVEC) cells, ADAM17 activity was required and modulated by extracellular sphingomyelinase for apoptosis induction [9]. Recently, ADAM17 activity has been linked to TNF mediated necroptosis induction in fibroblasts derived from ADAM17^ex/ex^ mice, showing reduced levels of ADAM17 [10]. We recently showed that the absence of ADAM17 resulted in reduced lung metastasis in a mouse model, due to a decrease in TNF mediated necroptosis in endothelial cells [11].

Here, we aimed to shed light on the roles of ADAM17 in different modes of TNF induced cell death in hematopoietic cells (U937 and Jurkat). Using the pharmacological inhibition of ADAM17 and genetic deficiency, achieved by CRISPR mediated knock-out of *ADAM17*, we show that ADAM17 depletion affects neither TNF-R1 surface expression, nor ligand mediated internalization nor TNF mediated NFκB induction. In U937, used as a model for monocytes, necroptosis is enhanced, whereas in the T-cell line, Jurkat apoptosis is enhanced while necroptosis is reduced. 

## 2. Materials and Methods

### 2.1. Reagents and Antibodies

Human TNF was a gift by D. Männel (University Hospital Regensburg, Germany). Biotinylated human TNF (BT210) and TL1a (1319-TL) were purchased from Bio-techne (Wiesbaden, Germany). KillerTRAIL (ALX-201-073-3020) was purchased from Enzo Life Sciences (Lörrach, Germany). FasL was produced as described previously [12]. All ligands were used at 100 ng/mL.

The inhibitors used in this study, TAPI-0 (#5523), GI254023X (#3995), Avagacestat (#6363), Necrostatin-1 (#2324) and GSK’872 (#6492), were purchased from Tocris (Tocris/bio-techne, Wiesbaden, Germany. zVAD (S7023) was purchased from Selleckchem. LY-411575 (SML0506-5MG) was purchased from Sigma-Aldrich. Cycloheximide (CHX) (#2112S) was purchased from Cell Signaling (Cell Signaling Technology, Frankfurt/M, Germany). All inhibitors were titrated and used at the highest non-toxic concentration: TAPI-0 (20 µM), GI254023X (7.5 µM), Necrostatin-1 (5 µM), GSK’872 (10 mM), Avagacestat (250 nM), Ly-411575 (1 µM), CHX (0.5 µg/mL), zVAD (50 µM).

Primary and secondary antibodies used for Western blot: anti-TACE/ADAM17 (ab2051 (Abcam, Berlin, Germany) and sc-390859 (Santa Cruz Biotechnology, Heidelberg, Germany)). Anti-cleaved caspase 3 (#9661S) anti-PARP1 (#9542S) and anti-MLKL (#14993S) were purchased from Cell Signalling (Cell Signaling Technology, Frankfurt/M, Germany). The HRP-conjugated anti-beta actin antibody was used as loading control (HRP-60008, Proteintech). Anti-mouse light chain HRP-conjugated antibody (AP200P) and anti-rabbit light chain HRP-conjugated antibody (MAB201P) were purchased from Merck/Millipore (Merck chemicals GmbH, Darmstadt, Germany).

Antibodies and controls used for flow cytometry analysis: anti-TNF-R1/CD120a-PE-Vio770 (130-127-505), anti-TNF-R2/CD120b-PE (130-124-000), anti-CD95-FITC (130-113-068). REA control-PE, REA control-PE-Vio70 (130-113-452), REA control-FITC (130-113-449) (Miltenyi Biotec, Bergisch Gladbach, Germany). 

### 2.2. Cell Culture

U937, Jurkat and MCF-7 cells were obtained from DSMZ (DSMZ-German Collection of Microorganisms and Cell Cultures GmbH, Braunschweig, Germany). The mEF cells were previously described [13]. Cells were cultivated in RPMI 1640 (U937 and Jurkat) or DMEM (MCF-7, mEF) (Gibco, Life Technologies, Darmstadt, Germany) and supplemented with 5% FCS (Gibco, Life Technologies, Darmstadt, Germany) and penicillin/streptomycin (Sigma-Aldrich, Taufkirchen, Germany). 

### 2.3. CRISPR/Cas9 Mediated Knock-Out

Knock-out was performed according to the Alt-R^®^ protocol, using IDT reagents (Alt-R^®^ S.p. Cas9 Nuclease V3 #1081058; Alt-R^®^ Cas9 Electroporation Enhancer #1075916; Alt-R^®^ CRISPR-Cas9 tracrRNA #1072533 and Alt-R^®^ CRISPR-Cas9 crRNA: Hs.Cas9.ADAM17.1.AA and Hs.Cas9.ADAM17.1.AB) (Integrated DNA Technologies, BVBA, Leuven, Belgium). RNP was transfected using the Amaxa nucleofector system (Lonza, Cologne, Germany). Single cell clones were derived by serial dilution followed by Western blot screening.

### 2.4. SDS-PAGE/Western Blot: IκB and Cell Death Assay, Whole Cell Lysate

To investigate IκB degradation or cell death induction by Western blot, 2–5 × 10^6^ cells were seeded and incubated with 100 ng/mL TNF for the indicated times. For cell death induction, CHX, zVAD or Nec1 was added as indicated, 30 min prior to addition of the ligand. Cells were harvested and total cell lysates were prepared using a modified RIPA buffer (50 mM TRIS-HCl [pH 7.5], 150 mM NaCl, 1% NP-40, 1% Triton X-100, 1 mM EDTA, 0.25% Na-deoxycholate), containing a protease inhibitor cocktail (Roche/Sigma-Aldrich, Taufkirchen, Germany). Protein concentration was determined by BCA (Pierce/Thermo Fisher Scientific, Schwerte, Germany). For the detection of MLKL under non-reducing conditions, DTT was omitted from the sample buffer. Samples were prepared immediately before SDS-PAGE. For SDS-PAGE, 12.5% PAA gels were used. Proteins were blotted onto a PVDF membrane (Carl-Roth, Karlsruhe, Germany). The membranes were blocked with 5% skimmed milk in TBST and incubated over night with the primary antibodies, diluted 1:1000 in 5% skimmed milk, as indicated in the respective figure legends. The peroxidase-conjugated secondary antibodies were incubated for 1 h, diluted 1:10,000 in 5% skimmed milk. Blots were developed using the LAS4000mini and ECL kit (RPN2236, Sigma-Aldrich, Taufkirchen, Germany).

### 2.5. Guava MUSE Cell Death Assay

For the assay, 5 × 10^5^–1 × 10^6^ cells were incubated with ligand (100 ng/mL; +/− CHX/zVAD) and inhibitors, as indicated in the respective figure legends, in 1 mL growth medium for 20 h at standard cell culture conditions. For staining, 25 µL of the cell suspension were mixed with 25 µL of the Annexin-V/7-AAD reagent provided in the kit (MCH100105) and incubated for 20 min in the dark, before 150 µL PBS was added. Data acquisition was performed using the Guava MUSE (Luminex, Austin TX, USA), according to the manufacturer’s instructions.

The adherent cells were seeded in 24 well plates at a density of 1 × 10^5^ z/well in 1 mL medium. After adhesion for at least 4 h, the cells were stimulated for 20 h and grown at standard cell culture conditions. To avoid loss of detached cells, the supernatant was stored prior to release of the cells with 100 µL Accutase (Gibco / Thermo Fisher Scientific, Schwerte, Germany)/well. All cells were pooled, sedimented and resuspended in 500 µL medium. For staining and measurement, 25 µL of this cell suspension was used, as described above, for suspension cells.

### 2.6. TNF-R1 Surface Expression, Internalization and ADAM17 Activity Assay

The surface expression of TNF-R1 was analysed by flow cytometry on U937 and Jurkat cells. Staining was performed using 5 × 10^5^ cells/assay. The cells were sedimented for 10 min at 300× *g* and cooled down on ice. Subsequently, the cells were stained by resuspending them in 98 µL staining buffer (PBS/2% BSA), supplemented with 2 µL fluorochrome-conjugated antibody for 10 min on ice. After being washed twice in PBS/BSA, the cells were finally resuspended in 150 µL PBS/2% PFA. 

For the analysis of ADAM17 activity, 5 × 10^5^ cells/assay were treated with 100 ng/mL PMA (79346, Sigma-Aldrich, Taufkirchen, Germany) for 3 h prior to receptor staining, as described above.

For the quantification of receptor internalization, 5 × 10^5^ cells/assay were harvested followed by washing in PBS/2% BSA and cooling down on ice for 15 min. Then 50 µL of PBS, containing 100 ng/mL biotinylated TNF (BT210, Bio-techne, Wiesbaden, Germany), was added and the cells were incubated for 15 min on ice. For internalization, the cells were transferred to a 37 °C water bath for 90 min followed by washing with 1 mL PBS/2% BSA and cooling down on ice for 15 min. The control cells were kept on ice to prevent receptor internalization but were also washed in 1 mL PBS/2% BSA after 90 min. The cells were then stained in 50 µL PBS/2% BSA containing 50 µL/mL Streptavidin-AlexaFluor647 (S32357, Thermo Fisher Scientific, Schwerte, Germany) for 30 min on ice. After being washed twice in 1 mL PBS/2% BSA, the cells were fixed in 2% PFA/PBS and analysed for receptor expression.

Flow cytometry was performed using a MACSquant Analyzer 10 (Miltenyi Biotec, Bergisch Gladbach, Germany). Data analysis was performed using FCSalyzer V0.9.22-alpha.

## 3. Results

### 3.1. Analysis of Cell Death Induction upon Pharmacological ADAM17 Inhibition

**Figure 1:**
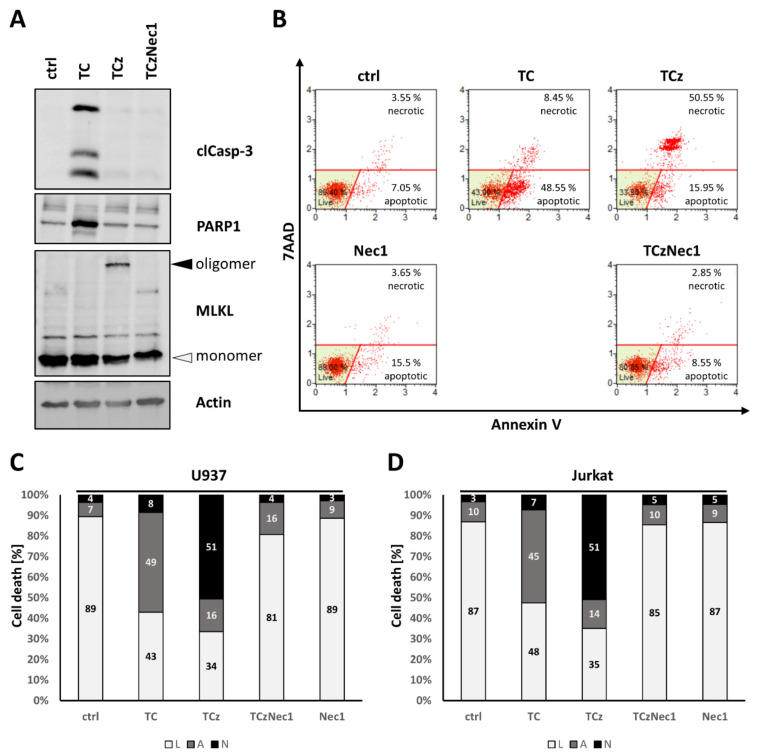
Analysis of cell death induction in human U937 and Jurkat cells. (**A**) depicts Western blot analysis of cell death induction in U937 cells. Untreated (control) cells were compared to cells treated with TNF/CHX (TC), TNF/CHX/zVAD (TCz) and TNF/CHX/zVAD/Nec1 (TCzNec1) for 4 h. The apoptosis marker proteins, cleaved Caspase-3 and PARP1, appeared upon TC treatment. Both disappeared when zVAD is additionally added (TCz) to shift cell death to necroptosis, which can be monitored by MLKL oligomerization (black arrowhead). In the presence of the pan-caspase inhibitor zVAD and the RIPK1 inhibitor Necrostatin 1, no cell death markers were activated. Actin served as loading control. (**B**) depicts cell death induction in U937 cells, quantified by flow cytometry analysis, using Annexin V/7AAD staining for apoptosis/necroptosis monitoring. Cells were incubated for 20 h with the indicated stimuli. Healthy, living cells are shown in lower left (green), apoptotic cells are shown in the lower right and necrotic cells are shown in the upper part of the scatter plot. (**C**) depicts data derived from flow cytometry analysis as bar graph for easier visualization of changes. Percentage of living cells (L) is shown in white, apoptosis (A) in grey and necroptosis (N) in black. (**D**) depicts cell death Induction in Jurkat T-cells. One representative of at least n = 3 experiments is shown.

In most cells, TNF triggers apoptosis upon the inhibition of protein synthesis via cycloheximide (CHX). Cell death shifts to necroptosis when caspases are additionally blocked, e.g., by the peptide inhibitor zVAD. Necroptosis, in turn, can be blocked by the addition of the RIPK1 inhibitor Necrostatin 1 (Nec1). Figure 1A shows Western blot (WB) analysis of cell death induction in U937 monocyte cells. Treatment with TNF/CHX (TC) leads to the cleavage of Caspase-3 and PARP1, whereas additional incubation with zVAD (TCz) results in oligomerization of the necroptosis marker MLKL (black arrowhead). Upon co-incubation with Nec1, neither cleaved Caspase-3, PARP1 nor MLKL oligomerization occurs. Cell death induction was quantified by flow cytometry, where apoptosis (48.55%) induction is indicated by increased Annexin V staining (Figure 1B, upper row, second panel). Uptake of the DNA intercalating dye 7-AAD occurs in both late apoptotic/necrotic cells (Figure 1B, upper row, second panel). In both U937 and Jurkat cells, Nec1 markedly reduced the percentage of Annexin-V^+^/7-AAD^+^ double positive necroptotic cells, indicating that necroptosis in these cells depends on RIPK1 activity.

The application of Nec1 was again used to reduce necrotic cell death from 55.5% (upper row, third panel) to 2.85% (lower row, right panel). For easier visualization, these data are depicted in Figure 1C as a bar diagram, and the same visualization is used in the following sections. Figure 1D shows the same analysis for Jurkat T-cells. Necroptosis induction can also be blocked in both U937 and Jurkat using the RIPK3 inhibitor GSK’872 (Figure S1A,B). 

**Figure 2:**
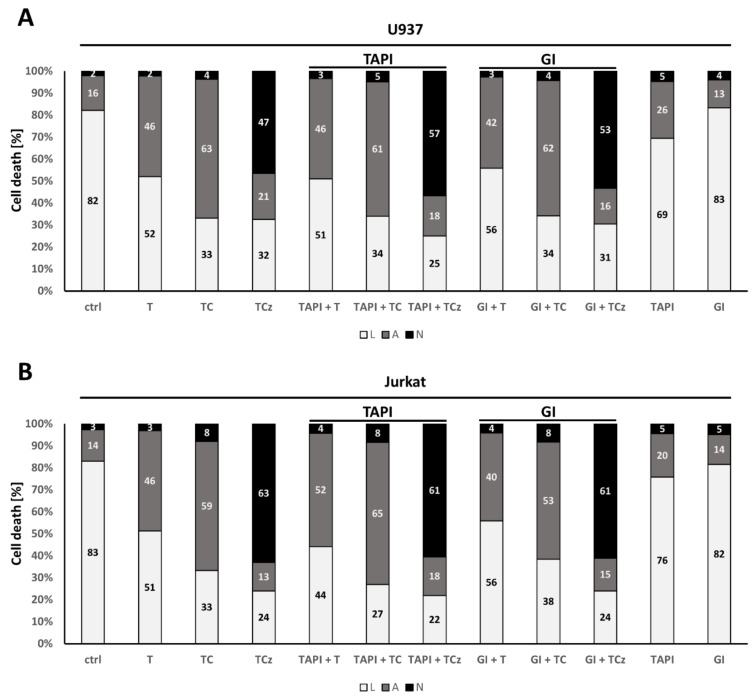
Analysis of cell death induction in human U937 and Jurkat cells upon pharmacological inhibition of ADAM17 and ADAM10. Quantification of cell death induction is depicted for (**A**) U937 and (**B**) Jurkat cells. Where indicated, cells were incubated for 20 h in the presence of the ADAM17 inhibitor TAPI-0 (TAPI) or the ADAM10 inhibitor GI254023X (GI). Both were added 30 min prior to addition of the death stimulus. The percentage of living cells (L) is shown in white, apoptotic (A) in grey and necroptotic (N) in black. One representative of at least n = 3 experiments is shown.

To investigate a role for ADAM17 in TNF mediated cell death induction in human myeloid cells, we incubated U937 and Jurkat cells with the ADAM17 protease inhibitor TAPI-0 prior to addition of T, TC or TCz. In U937 monocytes, TAPI did not affect T or TC mediated apoptosis, while necroptosis was slightly enhanced by ~10% (Figure 2A). THP-1 monocyte cells showed similar results (data not shown). In the Jurkat T-cell line no such effect could be observed (Figure 2B), indicating cell type-specific effects of ADAM17.

ADAM10 is a close relative of ADAM17 and is constitutively active. ADAM10 may compensate for the lack of ADAM17 activity [14]. To interrogate this, we incubated cells as described above with ADAM10- and ADAM17-dual specific inhibitor GI254023X. This did not affect apoptosis in either cell line analyzed. As for TAPI, in U937 cells necroptosis induction was slightly enhanced, whereas Jurkat cells showed no response at all (Figure 2A,C).

### 3.2. Knock-Out of ADAM17 Does Not Alter TNF-R1 Surface Expression and Internalization

**Figure 3:**
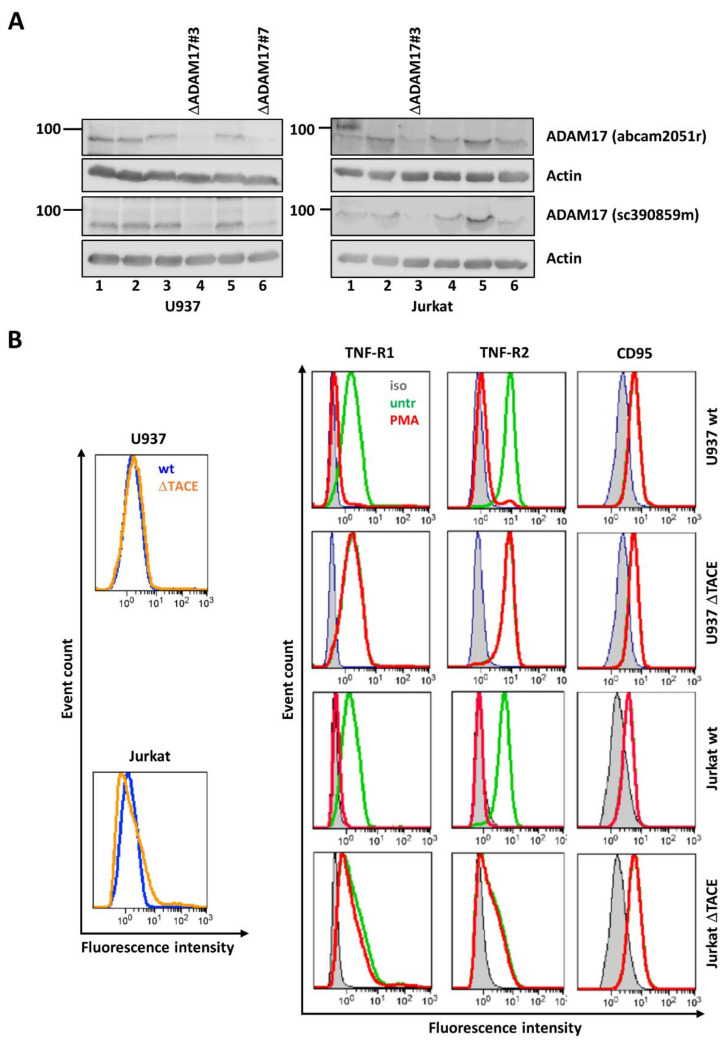

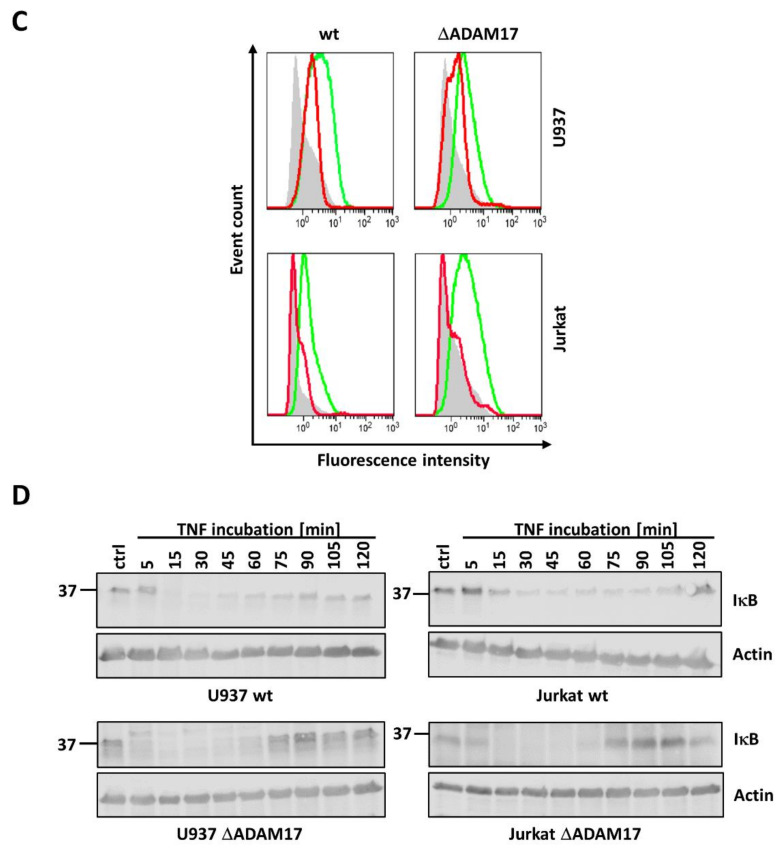
Validation of ADAM17 knock-out in U937 and Jurkat. (**A**) Western blot analysis of screening for ADAM17 knock-out cells (ΔADAM17) in U937 (left panels) and Jurkat (right panels) cells is shown. ADAM17 was probed using two different antibodies. ΔADAM17#7 cells were selected for U937, and clone ΔADAM17#3 was selected for Jurkat cells. Actin served as loading control. (**B**) Flow cytometry analysis of ADAM17 activity. The left panels depict analysis of TNF-R1 surface expression by flow cytometry: in both U937 and Jurkat wt (blue) vs. ΔADAM17 (orange) cells, the histogram curves are overall identical. Right panels: U937 or Jurkat cells were left untreated (green) or treated with PMA to trigger ADAM17 activity. The grey curve represents isotype (iso) controls. In wt cells the red curve shifts to the left, indicating shedding of TNF-R1 and TNF-R2 from the cell surface. In ΔADAM17 cells, the shift does not occur. CD95, which is not shed by ADAM17, serves as control, which is not shed by ADAM17. (**C**) Analysis of TNF induced TNF-R1 internalization. Cells without internalization are shown in green, cells upon 90 min TNF-R1 internalization are shown in red and unstained cells are shown in grey. U937 cells are shown in the upper two panels, Jurkat in the lower two panels. (**D**) Quantification of TNF induced IκB degradation in U937 wt (upper left) and ΔADAM17 (lower left), Jurkat wt (upper right) and ΔADAM17 (lower right). Actin served as loading control. For U937, ΔADAM17#3 was used in B-E. Representative results of at least n = 3 experiments are shown.

To guard against unspecific effects of small molecular inhibitors, we generated U937 and Jurkat cell lines, deficient for *ADAM17* expression, using CRISPR/Cas9 technology. Western blot analysis of the cell lysates with two different ADAM17-detecting antibodies showed nearly complete ADAM17 deficiency in two U937 cell clones (#3 and #7) and one Jurkat cell clone (#3) (Figure 3A). In both U937 and Jurkat wt and ΔADAM17#3 cells, the TNF-R1 surface expression was the same (Figure 3B, left panels). This rules out the possibility that the effects on signaling observed were due to altered TNF-R1 surface expression.

The lack of ADAM17 activity was validated by flow cytometry: we compared wt and ΔADAM17 cells before and after stimulation with the ADAM17 activating PMA [15]. This resulted in the shedding of TNF-R1, as well as TNF-R2 ectodomains from the cell surface in wt cells, while CD95 was not affected. The shedding of TNF-R1 and TNF-R2 was completely impaired in ΔADAM17 cells (Figure 3B, right panels). 

We previously showed that TNF-R1 mediated cell death induction requires TNF-R1 internalization in some cell lines [5,6]. Thus, the ΔADAM17 cells were compared to the respective wt cells regarding TNF-R1 internalization. The incubation of all cell lines with TNF for 90 min shows the internalization of TNF-R1 (Figure 3C). 

### 3.3. ADAM17 Knock-Out Does Not Affect TNF Induced NFκB Activation

The immediate and predominant response of most cell types towards TNF is the activation of NFκB signaling. To investigate the impact of ADAM17 knock-out on this pathway, the respective cell lysates were probed for the presence of IκBα to monitor its degradation upon receptor activation as a surrogate marker for NFκB activity. The onset of IκB degradation occurred in all cell lines ~15 min upon stimulation with TNF, irrespective of ADAM17 presence or absence (Figure 3D).

### 3.4. Genetic ADAM17 Deficiency Has Differential Effects on TNF Induced Cell Death in U937, Jurkat and mEF Cells

**Figure 4:**
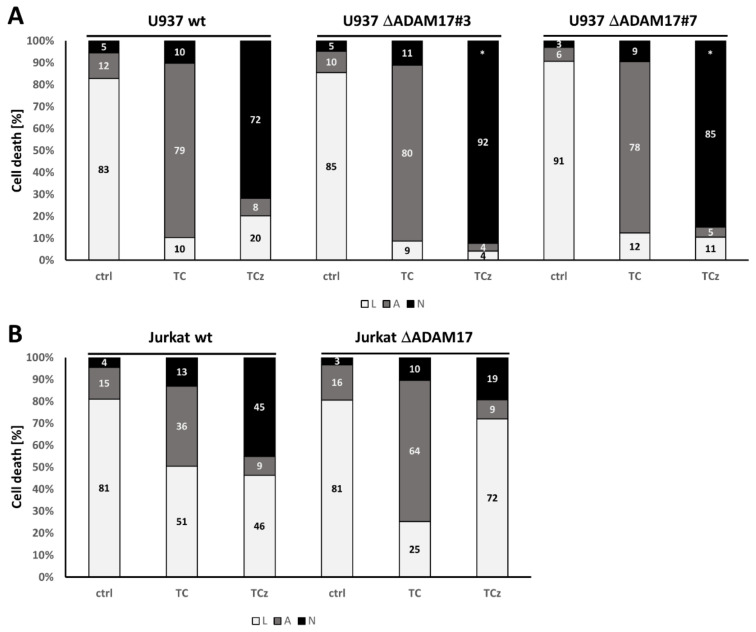

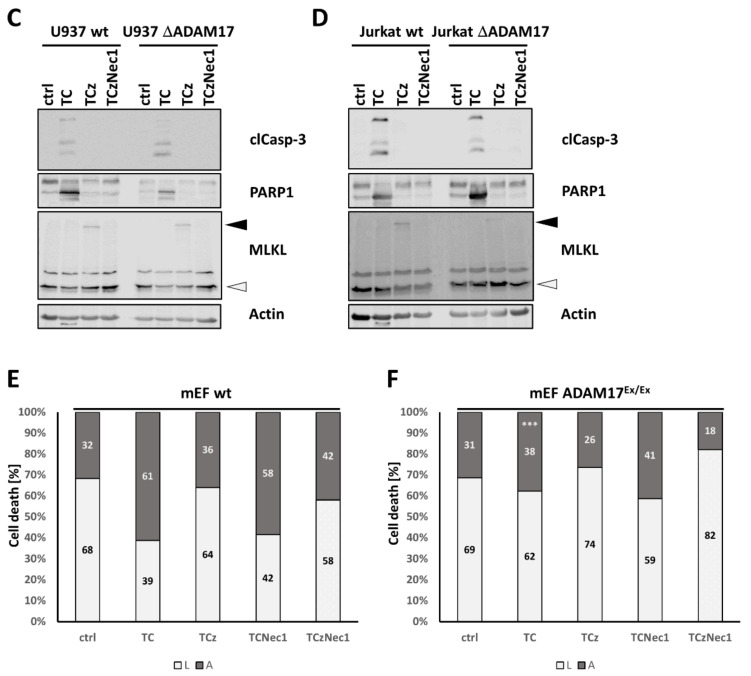
Analysis of TNF mediated cell death in wt versus ΔADAM17 cells. Cell death analysis in (**A**) U937 and (**B**) Jurkat cells, upon quantification by flow cytometry. The percentage of living (L, white), apoptotic (A, grey) and necroptotic (N, black) cells are shown. (**C**,**D**) depict Western blots probed for cleaved Caspase-3, PARP1 and MLKL, with actin as loading control. Where indicated, cells were incubated for 20 h in the presence of T, TC or TCz. (**E**,**F**) depict cell death induction in murine embryonic fibroblast cells derived from wt and animals with reduced ADAM17 levels (ADAM17^Ex/Ex^). We observed no necroptosis induction in either of the cell lines (see also Figure S1C); thus, only the percentages of living (L, white) and apoptotic (A, grey) cells are shown. Where applicable, statistical significance based on Student’s *t*-test is indicated (* *p* < 0.05; *** *p* < 0.001). Representative results of at least n = 3 experiments are shown.

We next analyzed TNF mediated cell death in wt and ΔADAM17 cells. The ablation of ADAM17 did not affect the apoptotic response of U937, while necroptosis induction was increased by 15–20% in knock-out cells (Figure 4A). In Jurkat cells, apoptotic cells were increased by ~30%, whereas the percentage of necroptotic cells was reduced by 50% in the absence of ADAM17 (Figure 4B). The Western blot analysis of cleaved Caspase-3, PARP1 and MLKL oligomerization was in line with the described results (Figure 4C,D).

In murine embryonic fibroblast cells (mEF) derived from wt mice, TC as well as TCz triggered only cell death, as indicated by the increased Annexin-V^+^/7-AAD^+^ double staining. In contrast to human cells, this could not be blocked by Nec1. Additional application of zVAD did not trigger necroptosis in these cells Figure 4E and Figure S1C, upper two rows). In order to analyze the impact of ADAM17 on cell death induction, we made use of mEFs derived from ADAM17^ex/ex^ hypomorphic mice, in which ADAM17 protein levels are reduced by 95% [13]. The apoptotic TNF response was, overall, reduced (Figure 4F and Figure S1C), showing that murine fibroblasts behave differently from human myeloid cells.

**Figure 5:**
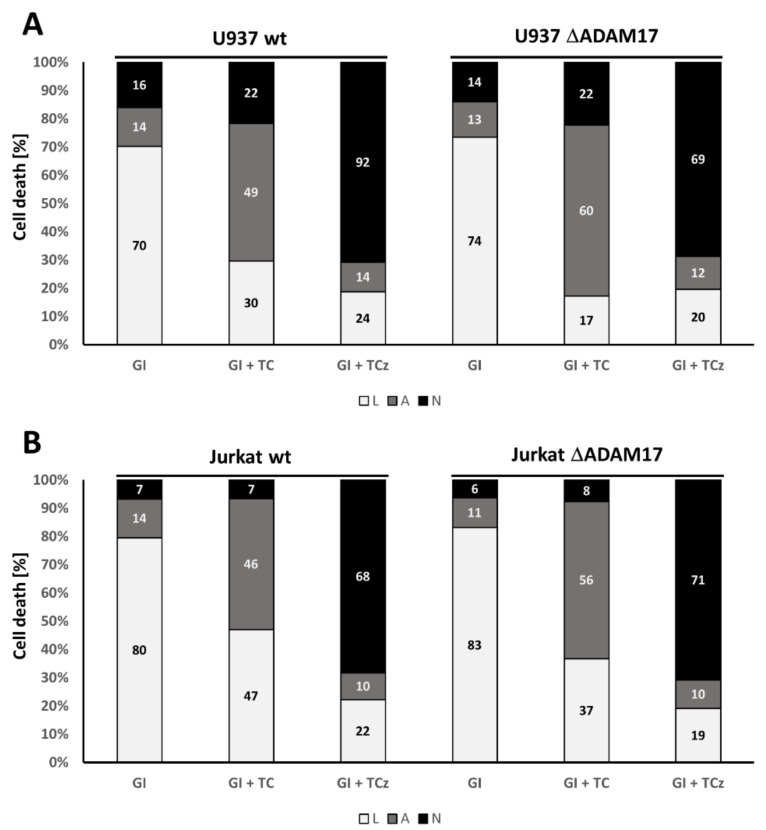
Analysis of ADAM10 in ΔADAM17 cells. Quantification of cell death is shown in (**A**) U937 wt vs. ΔADAM17 (clone 3) and (**B**) Jurkat cells. GI254023X (GI) was used to inhibit ADAM10 activity. The percentage of living (L, white), apoptotic (A, grey) and necroptotic (N, black) cells are shown. One representative of at least n = 3 experiments is shown.

To investigate a possible role of ADAM10 activity to compensate for the lack of ADAM17, we incubated ΔADAM17 cells with GI254023X, prior to stimulation with TNF. In U937 wt versus knock-out cells, the number of apoptotic cells was increased by 12% upon ADAM10 inhibition, whereas necrotic cells increased by ~24% (Figure 5A). In Jurkat cells, the effect on both types of cell death was negligible (Figure 5B). 

### 3.5. ADAM17 in the Regulation of DR3 Signal Transduction 

**Figure 6:**
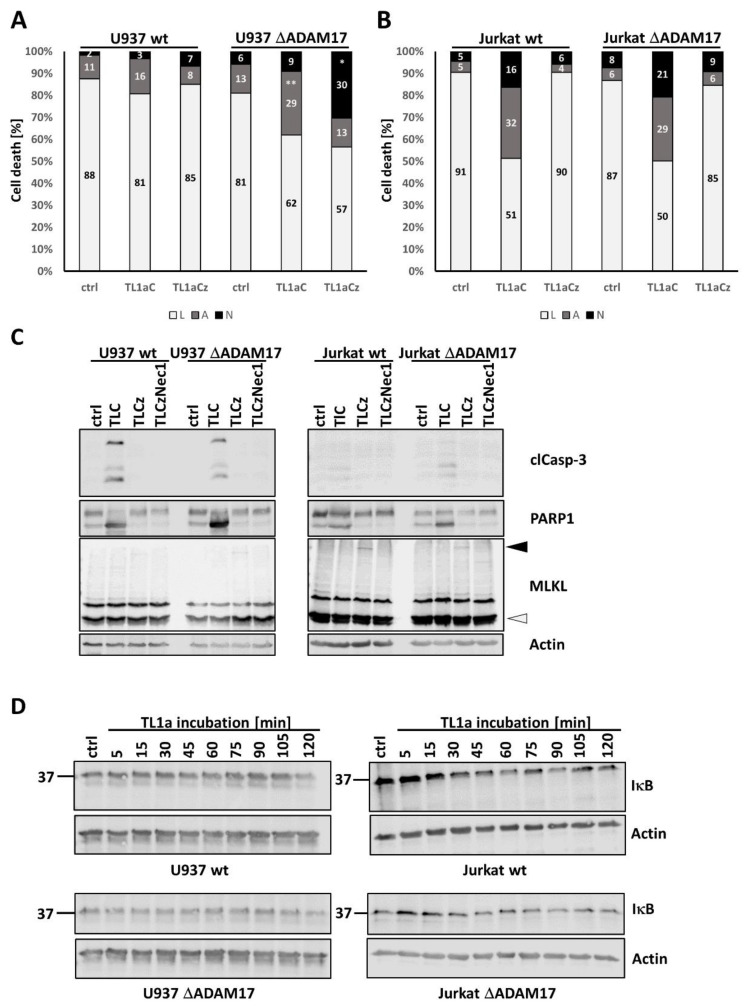
Analysis of ADAM17 and its role in DR3 signal transduction U937 and Jurkat cells. The DR3 ligand TL1a, in combination with CHX or CHC/zVAD, was used to induce cell death in (**A**) U937 and (**B**) Jurkat wt vs. ΔADAM17 cells. The percentages of living (L, white), apoptotic (A, grey) and necroptotic (N, black) cells are shown. (**C**) Western bot analysis of TL1a mediated cell death induction in U937 (left panels) and Jurkat (right panels) cells. Membranes were probed for cleaved Caspase 3, PARP1 and MLKL oligomers. Actin was used as loading control. (**D**) Western blot analysis of TL1a mediated IκB degradation, with Actin as loading control. In all settings, U937 ΔADAM17#3 was used. Where applicable, statistical significance based on Student’s *t*-test is indicated (* *p* < 0.05; ** *p* < 0.01). Representative results of at least n = 3 experiments are shown.

Signaling via death receptor 3 (DR3) is less well studied than the TNF-R1 system. The amino acid sequence of the receptor shows high similarity to TNF-R1. However, unlike TNF-R1, it is not ubiquitously expressed and, thus, is likely to signal differently [2,16]. In U937 wt cells, we observed moderate cell death induction upon DR3-stimulation with TL1a, while the absence of ADAM17 resulted in an increased percentage of apoptotic and necroptotic cells as assessed by flow cytometry analysis (Figure 6A). 

In Jurkat wt cells, the activation of DR3 by TL1a increased apoptosis in general by 23–28%. Simultaneously, the number of cells undergoing necroptosis was enhanced by 12–13% even without caspase-inhibition. The application of zVAD decreased both types of cell death. The ablation of ADAM17 did not alter TL1a induced apoptosis or necroptosis in Jurkat cells (Figure 6B). TL1a induced necroptosis was blocked by Nec1 incubation (not shown).

The Western blot analysis of TL1a treated U937 and Jurkat cells confirmed the flow cytometry data. Cleaved Caspase-3 and PARP1 appeared in all cell lines upon the addition of TL1a/CHX (TLC), whereas MLKL oligomers were barely detectable (Figure 6C).

Besides cell death, we also analyzed IκB degradation upon short-term TNF stimulation in these cells. TL1a did not trigger the degradation of IκB in any of the cell lines (Figure 6D). 

Overall, the signaling via DR3 was much weaker compared to TNF-R1 in U937 and Jurkat cells.

Cell death signaling via CD95 or TRAIL-R’s follows a similar scheme as it does for TNF-R1. However, by contrast, CD95 was not identified as a substrate for ADAM10/17. We quantified the cell death induction initiated by the respective ligands in U937 and Jurkat wt versus ADAM17 knock-out cells. In U937 cells the TRAIL (Tr) induced apoptotic response was reduced in ΔADAM17 cells by ~15%, whereas necroptosis was only mildly affected. FasL (Fl) induced cell death was not altered (Figure S2A). 

In Jurkat ΔADAM17 cells, TRAIL mediated apoptosis was reduced by ~20%. Similarly, necroptosis was reduced by ~17%. The same effect was observed for FasL mediated apoptotic or necroptotic cell death (Figure S2B). 

### 3.6. Pharmacological Inhibition of γ-Secretase Does Not Alter TNF-R1 Mediated Cell Death Induction

**Figure 7:**
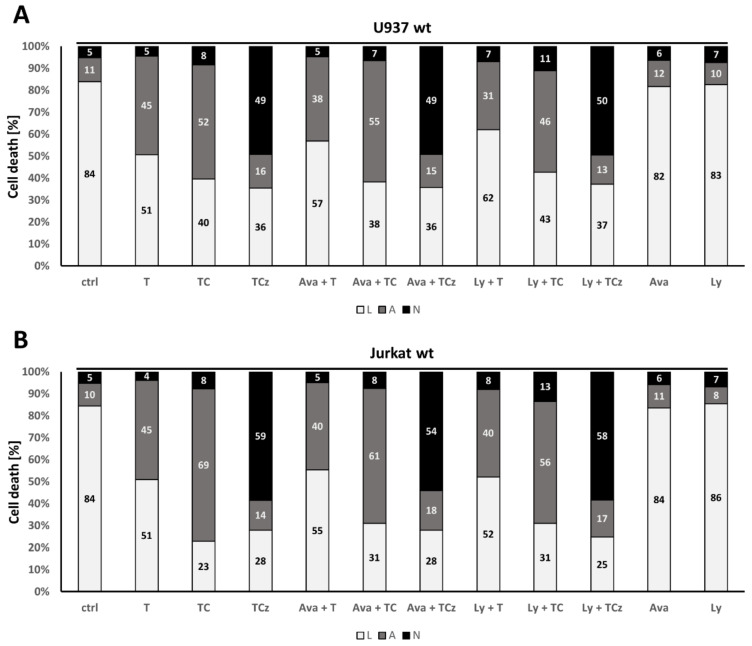

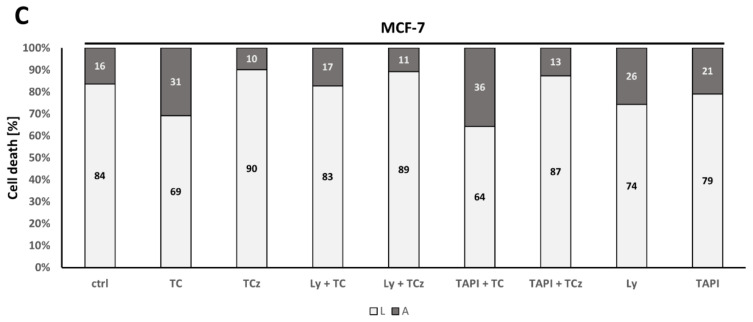
Analysis of the role of γ-secretase for cell death induction in U937 Jurkat and ADAM17 in MCF-7 cells. Flow cytometry based quantification of cell death triggered in (**A**) U937 and (**B**) Jurkat is shown. Where indicated, cells were incubated for 20 h in the presence of T, TC, TCz and the inhibitors of γ-secretase activity Avagacestat (Ava) and Ly-411575 (Ly). Inhibitors were added 30 min prior to addition of TNF. The percentages of living (L, white), apoptotic (A, grey) and necroptotic (N, black) cells are shown. (**C**) Cell death in MCF-7 cells was quantified in the presence of Ly and the ADAM17 inhibitor TAPI-0. The percentages of living (L, white) and apoptotic (A, grey) cells are shown, as MCF-7 cells do not undergo necroptosis due to their lack of RIPK3.

The ectodomain shedding by ADAM proteases is perceived as a prerequisite for the release of intracellular receptor domains (ICD) upon γ-secretase cleavage [8]. A role for γ-secretase in TNF mediated cell death induction in MCF-7 cells has been reported [7]. To investigate this effect in hematopoietic cells, we analyzed whether the pharmacological inhibition of γ-secretase complex activity by the inhibitors, Avagacestat and LY-411575, affected the outcome of cell death. Neither in U937 cells nor in Jurkat cells was the percentage of apoptotic or necroptotic cells altered (Figure 7A,B). However, in MCF-7 cells, the inhibition of the γ-secretase complex dampened TC mediated apoptosis induction from 30 to 17%, as previously described for this cell type. Interestingly, ADAM17-inhibition by TAPI did not affect induction of apoptosis in MCF-7 cells (Figure 7C). Additional zVAD treatment did not induce necroptosis, as MCF-7 cells are lacking *RIPK3* expression [17].

## 4. Discussion

The activation of TNF-R1 can induce diametrically opposed signaling cascades. NFκB signaling requires *complex I* formation at the plasma membrane, whereas cell death induction requires *complex IIa/b* or necrosome formation, resulting either in the induction of apoptosis or necroptosis, respectively [1]. Several reports showed that TNF mediated cell death induction requires full length TNF-R1 internalization for subsequent receptor bound *complex II* formation in different human and murine cell types and experimental settings [3,5,6,18,19,20,21,22,23]. These observations argue against a role for the shedding or RIPing of TNF-R1 in downstream cell death signaling in these systems. 

ADAM17/TACE was originally named the TNF alpha converting enzyme, based on its ability to release soluble TNF from cells by shedding. Meanwhile a plethora of other substrates (e.g., TNF-R1 or TNF-R2) were identified, and multiple biological functions have been described [14]. Thus, we asked whether the ADAM17 mediated, proteolytic processing of TNF-R1 is involved in TNF signaling in U937 monocytes, as well as in Jurkat T-cells.

**Figure 8:**
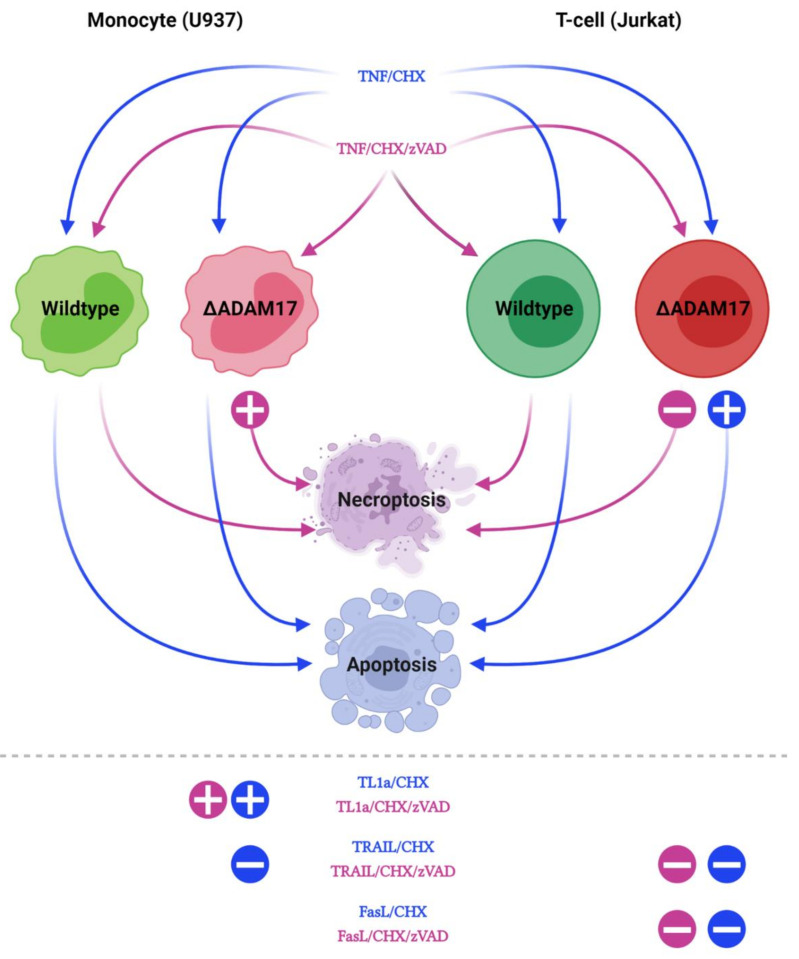
Model. The role of ADAM17 in TNF induced apoptosis (blue arrows) and necroptosis (purple arrows) was analyzed in U937 monocytes (**left**) and Jurkat T-cells (**right**). Increased or decreased cell death in knock-out (red) compared to the respective wt (green) cells is indicated by + or −. Below the dashed line, results for TL1a, TRAIL and FasL are shown.

The essence of our observations is depicted in Figure 8. The genetic ablation of ADAM17 enhances TNF induced necroptosis in U937 cells, whereas apoptosis is not affected. In Jurkat cells an ADAM17 deficiency reduces TNF mediated necroptosis and enhances the apoptotic response. TL1a mediated apoptosis and necroptosis are enhanced in U937 cells lacking ADAM17. TRAIL mediated apoptosis is reduced in U937 and Jurkat cells; TRAIL mediated necroptosis is also reduced in Jurkat cells. Both types of FasL mediated cell death are reduced in Jurkat cells lacking ADAM17, while U937 cells are not affected.

Comparing wild type mEFs and mEFs from ADAM17^ex/ex^ hypomorphic mice we observed a reduced apoptotic response. However, necroptosis could not be detected in either wt cells or cells lacking ADAM17. This observation is in contrast to an earlier report showing that ADAM17 deficient mEF cells were protected from TNF induced necroptosis [10]. In a more recent report, we revealed that the inhibition of ADAM17 blocks metastasis formation in a mouse model by abrogating necroptosis induction in endothelial cells. This required ADAM17 mediated TNF-R1 ectodomain shedding, followed by γ-secretase cleavage [11]. 

Here, we also showed that TNF-R1 RIPing by γ-secretase is not required for TNF induced cell death induction in hematopoietic cells. This contrasts with another earlier report showing that the activity of γ-secretase is required for TNF mediated TNF-R1 internalization and apoptosis induction in MCF-7 breast cancer cells [7]. It is generally assumed that ICD generation by γ-secretase intramembrane proteolysis requires ectodomain shedding by metalloproteases (i.e., ADAM17). In MCF-7 cells, however, necroptosis cannot be induced as they are lacking RIPK3, which is an essential necrosome component [17]. Liu and Chang reported reduced TNF shedding and, therefore, reduced autocrine TNF mediated NFκB activation upon piceatannol-treatment in U937 cells [24]. The activation of NFκB signaling represents the immediate early response to TNF in most cell types. We detected no alterations in NFκB activation upon ADAM17 depletion towards exogenous TNF. 

As has been frequently observed, the presented data, together with earlier reports by our and other groups, reveal that there are clear differences between cell lines of different provenance [1,2]. During metastasis formation, ADAM17 is required to trigger necrotic cell death in endothelial cells via TNF in order to allow the extravasation of tumor-derived cells. In monocytes and T-cells, the function of ADAM17 differs. Interestingly, the response towards other distinct ligands (TL1a, TRAIL or FasL) of the TNF superfamily also differs. The exact underlying molecular mechanism behind these differences remains to be elucidated in the future. A possible means of mediating such differences is presented by the differential use of lipids and the spatial membrane composition, and thus the organization of the micro-domains of membranes (i.e., raft vs. non-raft), in distinct types of cells. In particular, the activation of ADAM proteases and of members of the TNF-R1 superfamily has been frequently linked with localization to distinct membrane micro-domains [2].

In future, it will be of utmost interest to unravel the molecular basis (i.e., post-translational modification, subcellular localization or the membrane lipid environment) for such differences. Understanding the discrepancies may, in the future, allow us to define novel therapeutic strategies for the treatment of cancer, inflammatory diseases or infectious diseases. The present study has been performed using cancer cell lines; with this in mind, the obtained results should be tested in primary human cells, such as peripheral blood monocytes and lymphocytes. In the future, it will also be interesting to test how endogenous TNF production and release by monocytes are affected by ADAM17 knock-out. Such knock-out will result in enhanced levels of membrane bound TNF. This, in turn, can additionally activate TNF-R2 in an autocrine fashion and thereby trigger its capacity to cross-talk with TNF-R1 signaling through the activation of non-canonical NFκB signaling. Upon validation, the targeting of ADAM17 may be exploited to prevent tumor metastasis, while the enhanced apoptotic response of T-cells lacking ADAM17 could be facilitated to reduce the quantity of aberrant T-cells in leukemia, autoimmunity and inflammatory diseases. 

## Data Availability

Data is contained within the article or Supplementary Material.

## Supplementary Materials

The following are available online at https://www.mdpi.com/article/10.3390/cells10113100/s1, Figure S1: Inhibition of necroptosis by the RIPK3 inhibitor GSK’872 and cells death in mEF cells. (A,B) depict quantification of TC, TCz and TCz in the presence of GSK’872 in U937 and Jurkat cells. GSK was added 30 min prior to addition of TNF. The percentage of living (L, white), apoptotic (A, grey) and necroptotic (N, black) cells is shown. (C) Flow cytometry based analysis of cell death induction in mEF cells. Raw data corresponding to Figure 1E,F in wt mEF (panels, first row) TC shifts cells to the upper right quadrant of, which cannot be reverted by addition of Nec1. Thus, these cells are denoted as apoptotic. Additional treatment with zVAD does reduced the amount of dying cells, which is not altered upon co-treatment with Nec1 (panels, second row). In mEF derived from animals with reduced ADAM17 levels, cell death is reduced compared to wt mEF cells. zVAD and Nec1 have no additional effects (panels, third and fourth row), Figure S2: Analysis of FasL and TRAIL mediated cell death induction in U937 and Jurkat wt vs. ΔADAM17 cells. Cell death induction via TRAIL (Tr) and FasL (Fl) is depicted for (A) U937 wt (left) vs. ΔADAM17 (right) cells and (B) Jurkat wt (left) vs. ΔADAM17 (right) cells. The percentage of living (L, white), apoptotic (A, grey) and necroptotic (N, black) cells is shown.
