# Supplementary figures and images for: Roles for ADAM17 in TNF-R1 Mediated Cell Death and Survival in Human U937 and Jurkat Cells
Source: Cells. 2021 Nov 10;10(11):3100. doi: 10.3390/cells10113100 (PMC8620378; doi:10.3390/cells10113100)

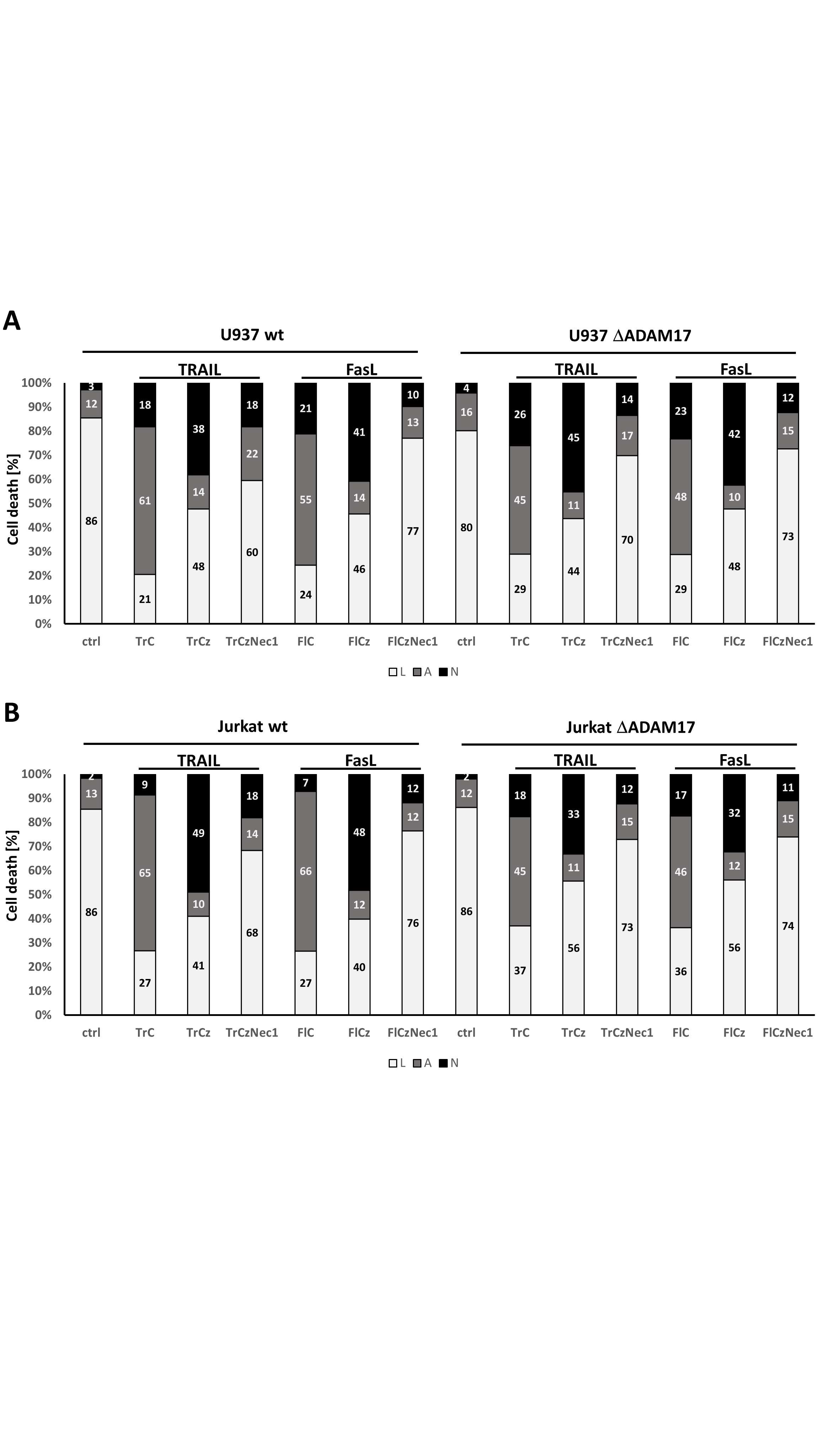

Supplement: Supplementary file 1 [file cells-10-03100-s001.zip › Figure S2A+B.TIF]

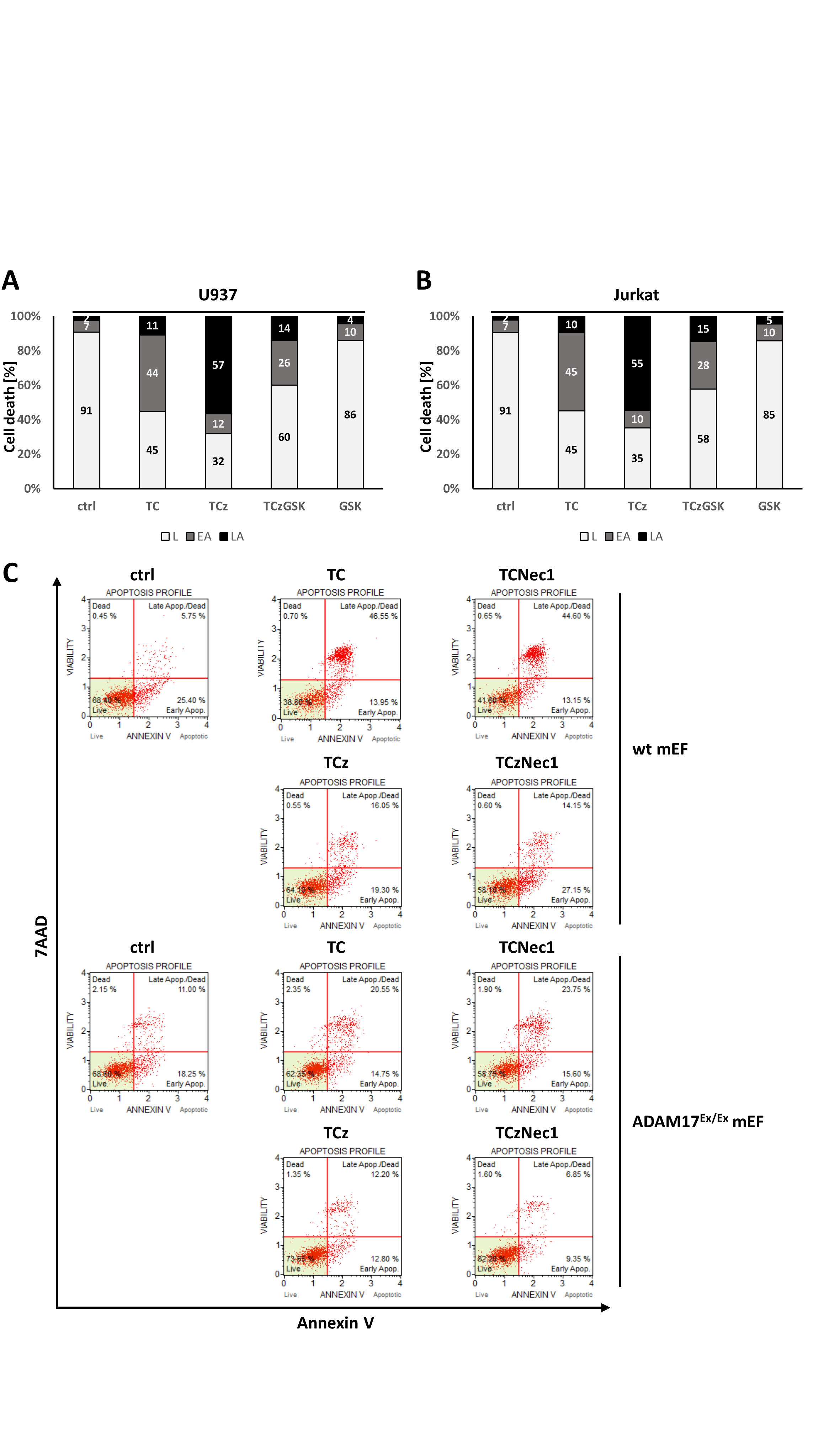

Supplement: Supplementary file 1 [file cells-10-03100-s001.zip › Figure S1A-C.TIF]
